# Morphological and molecular analysis of the tropical hermit crab *Calcinus vachoni* (Decapoda, Diogenidae) and its potential association with colonial anemone

**DOI:** 10.7717/peerj.15691

**Published:** 2023-07-25

**Authors:** Jibom Jung, Joong-Ki Park

**Affiliations:** Division of EcoScience, Ewha Womans University, Seoul, Republic of Korea

**Keywords:** *C. vachoni*, mtDNA cox1, Genetic divergence, Cryptic lineages, Ecological association, *Palythoa* aff. *mutuki*

## Abstract

*Calcinus* is the colorful hermit crab genus belonging to the family Diogenidae and is often found in coral reefs of the tropical Indo-West Pacific region, including southern Honshu, Japan, which is the northern limit of their occurrence. In the present study, we found *C. vachoni* for the first time in the intertidal zone of Jeju Island—the southernmost island of South Korea. We examined their morphology and provided a diagnosis of their morphological details with illustrations. In addition, the Korean *Calcinus* population was genetically characterized using mtDNA *cox1* sequences and by placing them into three previously reported regional haplogroups. The phylogenetic tree from maximum likelihood analysis revealed that Korean *C. vachoni* is assigned to the *C. vachoni* haplogroups exclusively, one of the three well-supported mitochondrial haplogroups with distinct geographic ranges (*i.e*., *C*. *vachoni*, *C*. aff. *vachoni* Cooks, and *C*. aff. *vachoni* Mascarenes). This result provides new information on the species distribution of *C.vachoni*, extending their geographic range further north into the southern coast of Korea. In this study, we also first report the potential association of *C. vachoni* with their co-occurring colonial anemone species *Palythoa* aff. *mutuki* and dead coral head of *Pocillopora* species based on our on-site observation and a public coral collection database of *Calcinus* species. However, their ecological association with co-occurring coral species is putatively assumed for now and therefore has to be validated by compelling evidence from further field observation and experimental studies (*i.e*., whether the presence/absence of colonial anemones affects the behavior and survival of the hermit crabs).

## Introduction

The family Diogenidae Ortmann, 1892 is one of the seven taxa in the superfamily Paguroidea Latreille, 1802 and consists of 22 genera (Lemaitre & McLaughlin, 2023b). They are found in various habitats, from intertidal to slightly deep subtidal, and most species are found in empty shells. Some species are known to show a symbiotic association with anemones. Members of this family are readily distinguished from other hermit crabs by their third maxillipeds that are close to each other in their bases. This family is also characterized by other characteristics: the left cheliped is generally larger than the right one, the antennular flagella terminate in a filament, no paired pleopods on the fourth and fifth abdominal somites, and the abdominal tergites are mostly not well calcified (McLaughlin, 2003). Among the genera of the Diogenidae, genus *Calcinus* is mostly moderate-to-large in body size and have 13 pairs of gills, left cheliped larger than the right one, moderate to well-developed triangular rostrum, and colorful carapace and pereopods (McLaughlin, 2003; McLaughlin et al., 2007). The color pattern of the *Calcinus* species is considered highly useful for distinguishing the species (McLaughlin et al., 2007; Malay & Paulay, 2010). Overall, 47 *Calcinus* species have been reported worldwide (McLaughlin et al., 2010; Lemaitre & McLaughlin, 2023a); most of these are found in the tropical or subtropical coral reefs in the Indo-West Pacific region (Malay & Paulay, 2010). They are considered to show a facultative symbiotic association with a wide range of coral species including *Acanthastrea echinata, Pocillopora grandis, P. verrucosa, P. meandrina, Seriatopora hystrix, Acropora, Montipora, Porites*, and *Xenia* spp. (Hazlett & Bach, 2010; Malay & Paulay, 2010; Britayev & Mikheev, 2013; Florida Museum Invertebrate Zoology Collection (http://specifyportal.flmnh.ufl.edu/iz/)). Nevertheless, a comprehensive phylogenetic and biogeographic analysis for the genus *Calcinus* species using multi-locus genetic information (mtDNA *cox1* and 16S rDNA, and histone three nuclear DNA sequences) uncovered a wealth of hidden diversity of *Calcinus* species in which *C. vachoni* was subdivided into three cryptic lineages (*i.e*., *C*. *vachoni*, *C*. aff. *vachoni* Cooks, and *C*. aff. *vachoni* Mascarenes), each showing allopatric distribution (Malay & Paulay, 2010).

In the faunal study of hermit crabs in Korea, we found *C. vachoni* for the first time in the intertidal zone of Jeju Island—the southernmost island of South Korea. In the present study, we examined their morphology and provided a diagnosis of their morphological details with illustrations. We also conducted a molecular identification of the Korean *C. vachoni* specimens using the mtDNA *cox1* sequences by comparing them with the previously published sequences available on GenBank that includes the sequences of Malay & Paulay (2010). In addition, we report the potential association of *C. vachoni* with the colonial anemone species *Palythoa* aff. *mutuki* for the first time based on on-site observation of their habitat, literature review and a public collection database of *Calcinus* species.

## Materials and Methods

**Figure 1:**
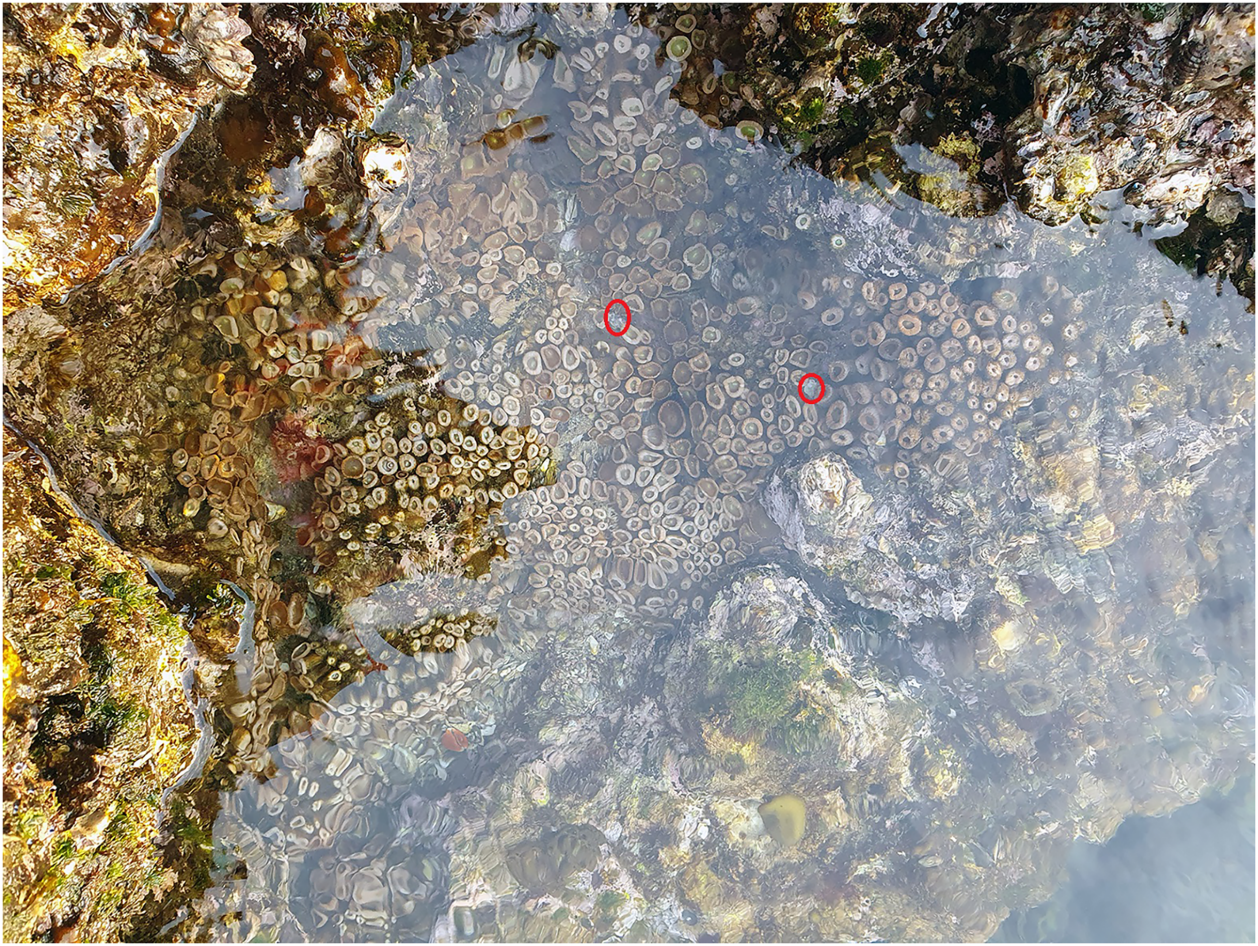
A tide pool where *Calcinus vachoni* was found co-occurring with the colonial anemone, *Palythoa* aff. *mutuki*. Red circle: *Calcinus vachoni*.

Twelve individuals of *C. vachoni* were collected from nearby living colonial anemones (Fig. 1) in the rocky intertidal zone of Gamsan-ri, Andeok-myeon, Seogwipo-si, Jeju Island (33°14′07.1″N 126°21′31.4″E), the Republic of Korea, on September 6, 2021. We determined ITS1-5.8S-ITS2 sequences and the partial sequence of 28S rDNA (GenBank accession nos.: OQ456449--OQ456456) for species identification of colonial anemones. Sequence comparison and BLAST search for our colonial anemone samples were not able to distinguish *P*. aff. *mutuki* from *P*. *mutuki* because the nucleotide sequences between these two species were very similar (95.4–98.4%), not separated in the previous analysis (Mizuyama, Masucci & Reimer, 2018). Nevertheless, the morphological characteristics of our samples agree with *P*. aff. *mutuki* in that they have a smooth capitular ridges surface (Fig. 1) consistent with Mizuyama, Masucci & Reimer (2018), differing them from *P. mutuki* with a jagged surface.

To investigate potential factors affecting the habitat of *C. vachoni*, a qualitative survey of on-site observation was conducted on 12 studied sites in tidepool areas within 500 m of their habitat (Table 1). Voucher specimens were deposited in the Honam National Institute of Biological Resources (HNIBR voucher specimen no.: HNIBRIV911). All specimens were fixed in 95% ethanol and subjected to morphological and molecular analysis. Morphological characteristics were examined using an MZ8 dissection microscope (Leica, Wetzlar, Germany). The shield length was measured as the body size from the tip of the rostrum to the midpoint of the posterior margin of the carapace using a CD6CSX digital caliper (Mitutoyo, Kawasaki, Japan) at the 0.1-mm scale. The main focus of the present study is to characterize morphological and molecular information of the Korean *Calicinus* species. Therefore, our taxon sampling for these analyses is limited to the Korean population, along with some other Micronesian *Calicinus* species as reference taxa (Table 2).

**Table 1:** Detailed information on the 12 surveyed sites.

Site	GPS	Presence/absence of *Calcinus vachoni*	Presence/absence of *Palythoa* aff. *mutuki*
Site #1	33°14′07.1″N 126°21′31.2″E	O	O
Site #2	33°14′07.4″N 126°21′31.5″E	X	O
Site #3	33°14′08.1″N 126°21′34.1″E	X	X
Site #4	33°14′10.3″N 126°21′32.7″E	X	X
Site #5	33°14′09.8″N 126°21′36.1″E	X	X
Site #6	33°14′09.2″N 126°21′34.9″E	X	X
Site #7	33°14′08.4″N 126°21′35.6″E	X	X
Site #8	33°14′06.6″N 126°21′34.1″E	X	X
Site #9	33°14′06.5″N 126°21′35.1″E	X	X
Site #10	33°14′06.3″N 126°21′36.3″E	X	X
Site #11	33°14′05.7″N 126°21′36.4″E	X	X
Site #12	33°14′05.3″N 126°21′37.8″E	X	X

**Note:**

O, presence; X, absence.

**Table 2:** GenBank accession numbers and geographic information of Decapoda species used for phylogenetic analysis in this study.

Family	Species	Location	Specimen number	mtDNA *cox1* GenBank accession no
Calcinidae	*Calcinus vachoni*	Jeju, Korea	HNIBRIV911	**ON763592**
				**ON763593**
				**ON763594**
				**ON763595**
				**ON763596**
		Balingasay, Philippines	UF 6748	FJ620291
		Maug Island, Mariana Islands	UF 5742	FJ620339
		Okinawa, Japan	UF 6992	FJ620395
		China		MK747767
	*Calcinus* aff. *vachoni* Mascarenes	Reunion Island, Mascarene Islands	UF 12634	FJ620293
			UF 13011	FJ620294
				FJ620295
		Sodwana Bay, South Africa	MB-A066068	MH482034
			MB-A065989	MH482078
		Port Shepstone, South Africa	MB-A066419	MH481935
			MB-A066420	MH482017
		Pumula, South Africa	MB-A066399	MH481962
			MB-A066397	MH482022
			MB-A066398	MH482045
	*Calcinus* aff. *vachoni* Cooks	Rarotonga Island, Cook Islands	UF 1377	FJ620296
			UF 11702	FJ620292
	*Calcinus elegans*	Kosrae, Micronesia	MADBK 160518_004	**ON763555**
			MADBK 160518_006	**ON763556**
				**ON763557**
		Rangiroa Atoll, Tuamotu Archipelago	UF 1351	FJ620284
	*Calcinus guamensis*	Kosrae, Micronesia	MADBK 160535_002	**ON763558**
				**ON763559**
		Hiva Oa Island, Marquesas Islands	UF 5171	FJ620288
	*Calcinus haigae*	Kosrae, Micronesia	MADBK 160534_004	**ON763560**
				**ON763561**
				**ON763562**
		American Samoa	UF 3225	FJ620307
	*Calcinus laevimanus*	Kosrae, Micronesia	MADBK 160519_016	**ON763563**
				**ON763564**
		Reunion Island, Mascarene Islands	UF 5426	FJ620270
	*Calcinus lineapropodus*	Kosrae, Micronesia	MADBK 160524_007	**ON763565**
				**ON763566**
				**ON763567**
		Guam Island, Mariana Islands	UF 1322	FJ620255
	*Calcinus minutus*	Kosrae, Micronesia	MADBK 160536_005	**ON763568**
				**ON763569**
		American Samoa	UF 3263	FJ620303
	*Calcinus morgani*	Kosrae, Micronesia	MADBK 160530_001	**ON763570**
		American Samoa	UF 3236	FJ620277
	*Calcinus pulcher*	Kosrae, Micronesia	MADBK 160537_003	**ON763571**
				**ON763572**
				**ON763573**
		Pohnpei Island, Micronesia	UF5396	FJ620377
Diogenidae (outgroup)	*Dardanus deformis*	Kosrae, Micronesia	MADBK160523_007	**ON763584**

**Note:**

Accession numbers in bold: sequences obtained from this study.

For molecular analysis, the first or second ambulatory legs of five Korean *C. vachoni* specimens were excised for total genomic DNA extraction using DNeasy Blood & Tissue Kits (Qiagen, Hilden, Germany). For molecular identification of *Calcinus* specimens, we performed phylogenetic analysis of mtDNA *cox1* sequences obtained from 19 individuals of eight *Calcinus* species collected from South Korea and Micronesia that were newly sequenced in this study (voucher specimens of Micronesian hermit crabs have been deposited in the Marine Arthropod Depository Bank—MADBK; MADBK voucher specimen nos. are as follows: MADBK 160518_004, MADBK 160518_006, MADBK 160519_016, MADBK 160524_007, MADBK 160530_001, MADBK 160534_004, MADBK 160535_002, MADBK 160536_005, and MADBK 160537_003; Table 1). To amplify the mitochondrial *cox1* gene fragment, the universal primers LCO1490 and HCO2198 were used (Folmer et al., 1994). Polymerase chain reaction (PCR) was performed using 3 µL of DNA template, 5 µL of 10 × Ex Taq buffer, 5 µL of dNTP mix (10 mM), 2 µL of each primer (10 µM), 0.25 µL of Go Taq DNA polymerase (Promega, Madison City, WI, USA), and 35.75 µL of distilled H_2_O to make up a total volume of 50 µL. PCR was conducted as per the following steps: 10 min denaturation at 94 °C, followed by 40 cycles of 1 min at 94 °C, 1.5 min at 45 °C, and 2 min at 72 °C and then a final extension for 10 min at 72 °C. PCR products were visualized on 1% agarose gels and sequenced using an ABI PRISM 3730xl DNA Analyzer (Applied Biosystems, Foster City, CA, USA). The nucleotide sequences of *cox1* were edited using the Geneious Prime software v.2022.0.1 (Biomatters, Auckland, New Zealand) and its Clustal Omega program (Sievers et al., 2011). The *cox1* sequences of the Korean *C. vachoni* specimens and the Micronesian samples were deposited in GenBank (MZ215675–MZ215720). In addition, 25 mtDNA *cox1* sequences of nine *Calcinus* species and an outgroup species (*Dardanus deformis*) were retrieved from GenBank and included in the phylogenetic analyses (Table 1).

Species identification of the Korean *C. vachoni* specimens was molecularly confirmed by reconstructing their phylogenetic relationships with other hermit crab species using maximum likelihood (ML) analysis of the *cox1* sequence data with the MEGA10 program (Kumar et al., 2018). The ML analysis of *cox1* sequences was performed based on the general time reversible models (Tavaré, 1986) with gamma distribution (+G) and invariable sites (+I) rate categories that were obtained from the Bayesian Information Criterion scores model using the jModelTest 2.1.7 application (Posada, 2008). The robustness of individual nodes in the ML trees was assessed using bootstrap analysis with 1,000 pseudo-replicates. Interspecific and intraspecific sequence divergences were estimated using the K2P distance matrix in the MEGA10 program.

## Results and Discussion

### Morphological characteristics of *C. vachoni*

The morphological characteristics of the Korean *Calcinus* specimens coincided with the original description of *C. vachoni* (Forest, 1958). The genus *Calcinus* is distinguishable from other hermit crab genera by the following morphological characteristics: 13 pairs of gills, left cheliped larger than the right one, absence of paired pleopods, and well-developed triangular rostrum. *Calcinus vachoni* is distinguishable from other congeneric species based on the following morphological characteristics: ventral margins of the dactyl and propodus of ambulatory legs have sparse tufts of setae; dorsal margin of the right chela has 3–6 tubercles; ventral margins of the dactyls and propodi of the second ambulatory legs have a higher number of tufts of setae compared with the margins of those of the first ambulatory legs; the telson exhibits numerous spines on the terminal to lateral margins; and the pereopods exhibit a cream dactyl and bluish-gray propodi. Since *C. vachoni* is currently divided into three haplogroups from molecular analysis (Malay & Paulay, 2010), *i.e*., *C. vachoni*, *C*. aff. *vachoni* Cooks, and *C*. aff. *vachoni* Mascarenes (Malay & Paulay, 2010), and Korean *Calcinus* specimens were identified as the *C. vachoni* haplogroup based on mtDNA *cox1* sequences (see detail in the Results & Discussion section). Morphological diagnosis of the *C. vachoni* haplogroup is first reported below.

SYSTEMATICS

Class Malacostraca Latreille, 1802

Order Decapoda Latreille, 1803

Family Diogeinidae Ortmann, 1892

Genus *Calcinus* Dana, 1851

**Figure 2:**
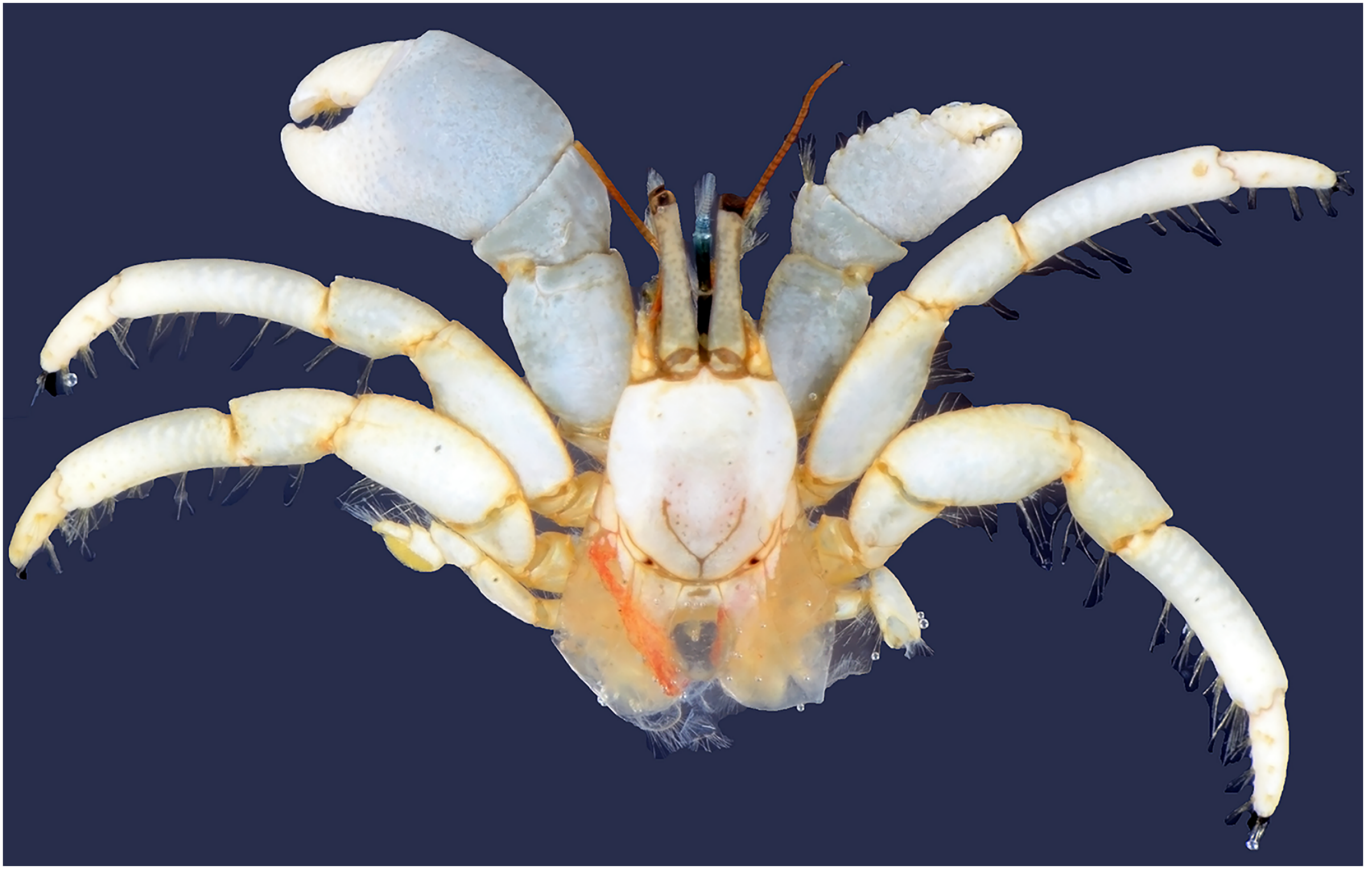
Dorsal view of *Calcinus vachoni* Forest, 1958 (male, shield length 3.5 mm, HNIBRIV911, abdomen lost).

**Figure 3:**
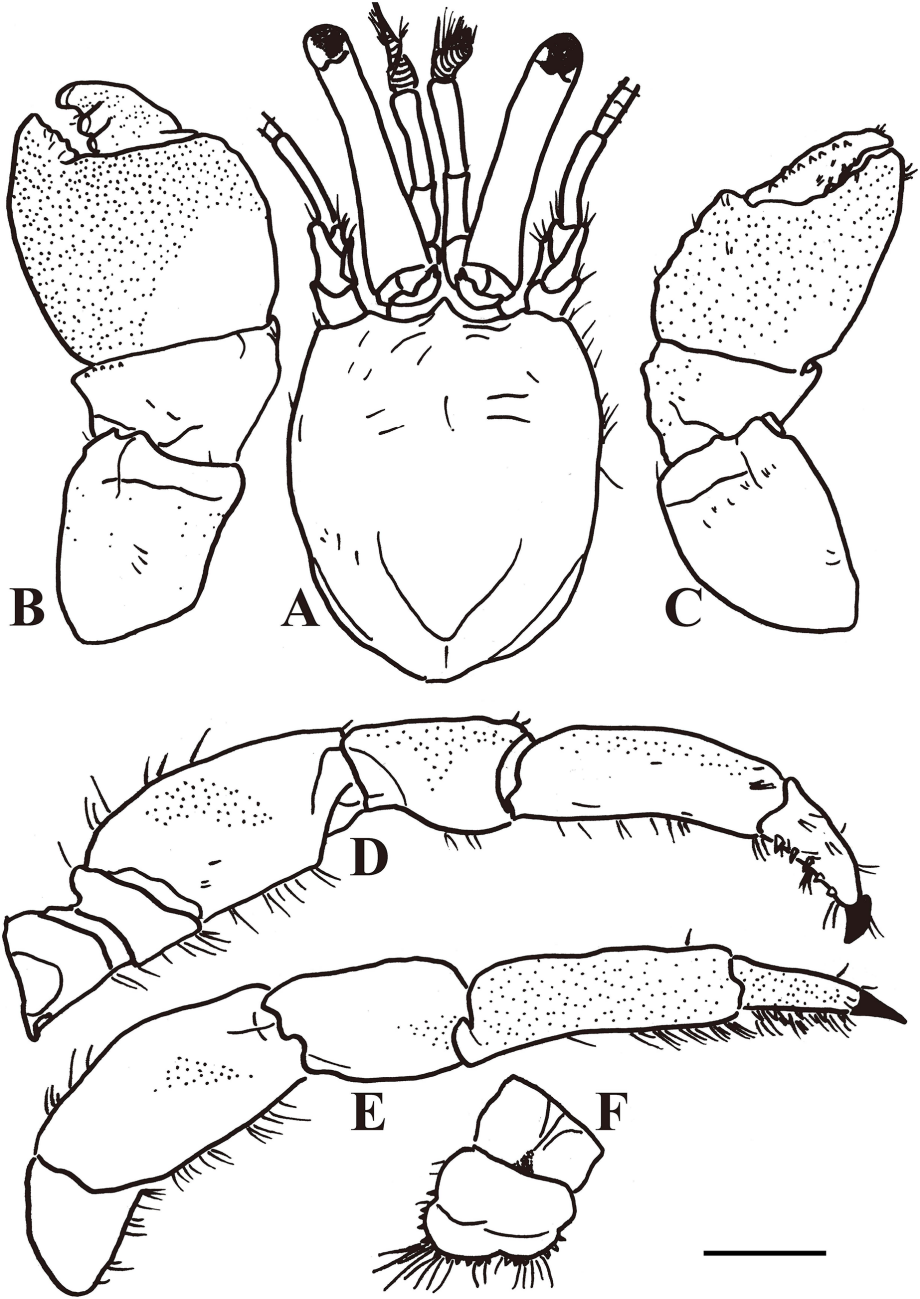
*Calcinus vachoni* Forest, 1958 (male, shield length 2.9 mm, HNIBRIV911). (A), Shield and cephalic appendages, dorsal view; (B), left cheliped, dorsal view; (C), right cheliped, dorsal view; (D), right first ambulatory leg, mesial view; (E), left second ambulatory leg, lateral view; (F), telson, dorsal view. Scale bar: 2 mm.

***Calcinus vachoni*** Forest, 1958 (Figs. 2 and 3).

*Calcinus vachoni* Forest, 1958: 285, Figs. 2, 3, 9, 10, 15, 19; Morgan, 1990: 11, Fig. 2; Morgan, 1991: 205, Figs. 60–62; Poupin, 1997: 712, Figs. 6E, F, 8A–F; McLaughlin et al., 2007: 170–171; McLaughlin et al., 2010: 19; Arima, 2014: 42.

*Calcinus seurati* Miyake, 1963: 63; Matsuzawa, 1977: pl. 79, Fig. 3; Miyake, 1983: 113; Chang & Chen, 1992: 108 (not *Calcinus seurati* Forest, 1951).

Not *Calcinus vachoni* Lewinsohn, 1982: 53 (= *Calcinus guamensis* Wooster, 1984).

Materials examined: A total of 12 individuals (shield length 2.6–3.9 mm), Gamsan-ri, Andeok-myeon, Seogwipo-si, Jeju Island, Korea (33°14′05″N, 126°21′30″E) rocky intertidal, nearby colony of *P*. aff. *mutuki*, 6 Sep 2021, coll. J Jung, HNIBRIV911.

Diagnosis: A total of 13 pairs of phyllobranchiate gills. Shield (Figs. 2 and 3A) semi-ellipse, 1.1 times longer than width; rostrum and lateral projections broad triangular. Ocular peduncle 0.8 shield length, inflated basally; cornea slightly dilated; ocular acicles terminally bi- or trifid. Antennular and antennal peduncles shorter than ocular peduncles when fully extended. Pereopods with numerous fine granules. Chelipeds unequal, left appreciable larger. Lateral and dorsal surfaces of palm of left cheliped (Figs. 2 and 3B) without distinct spines or tubercles. Right cheliped (Figs. 2 and 3C) with 3–6 tubercles on dorsal surface of palm; carpus with 2–3 small spines on dorsal margin. Ambulatory legs (Figs. 2, 3D, and 3E) with numerous fine granules, 2.7–3.3 times as long as shield. Ventral margins of dactyl, propodus, and meri with tufts of moderate setae, other margins with sparse setae or naked; dactyl 0.4–0.5 propodus length, ventral margin with 4–5 corneous spines. First ambulatory leg with fewer tufts of setae on ventral margins of dactyl and propodus than on those of second. Abdomen twisted, membranous. No paired pleopods in either sex, abdominal tergites not well calcified. Uropods asymmetrical. Telson (Fig. 3F) asymmetrical, left posterior lobe larger than right; terminal and lateral margins of posterior lobes with numerous spines.

Color: Shield creamy white. Ocular peduncles bluish-gray; acicles creamy white. Antennular peduncle deep blue. Antennal flagellum red. Chelipeds generally bluish-gray except cream fingers. Ambulatory legs cream or light bluish-gray; dactyl generally cream.

Distribution: Jeju Island, Korea (a new record of its occurrence); Vietnam (type locality); Indo-Pacific region from South Africa to Easter Island and southern Japan; shallow subtidal to 20 m (Forest, 1958; Murata, Watanabe & Asakura, 1991; Poupin, 1997; Poupin, Boyko & Guzmán, 2003; McLaughlin et al., 2007; Malay & Paulay, 2010; Landschoff & Gouws, 2018).

Habitat: found in living *P*. aff. *mutuki* in intertidal zone; nearby living and dead corals such as *P. verrucosa* and other *Pocillopora* spp. from subtidal to 20 m depth (McLaughlin et al., 2007; Arima, 2014; Florida Museum, 2023).

### Molecular phylogenetic analysis of Korean *C. vachoni* with some other *Calcinus* species

**Figure 4:**
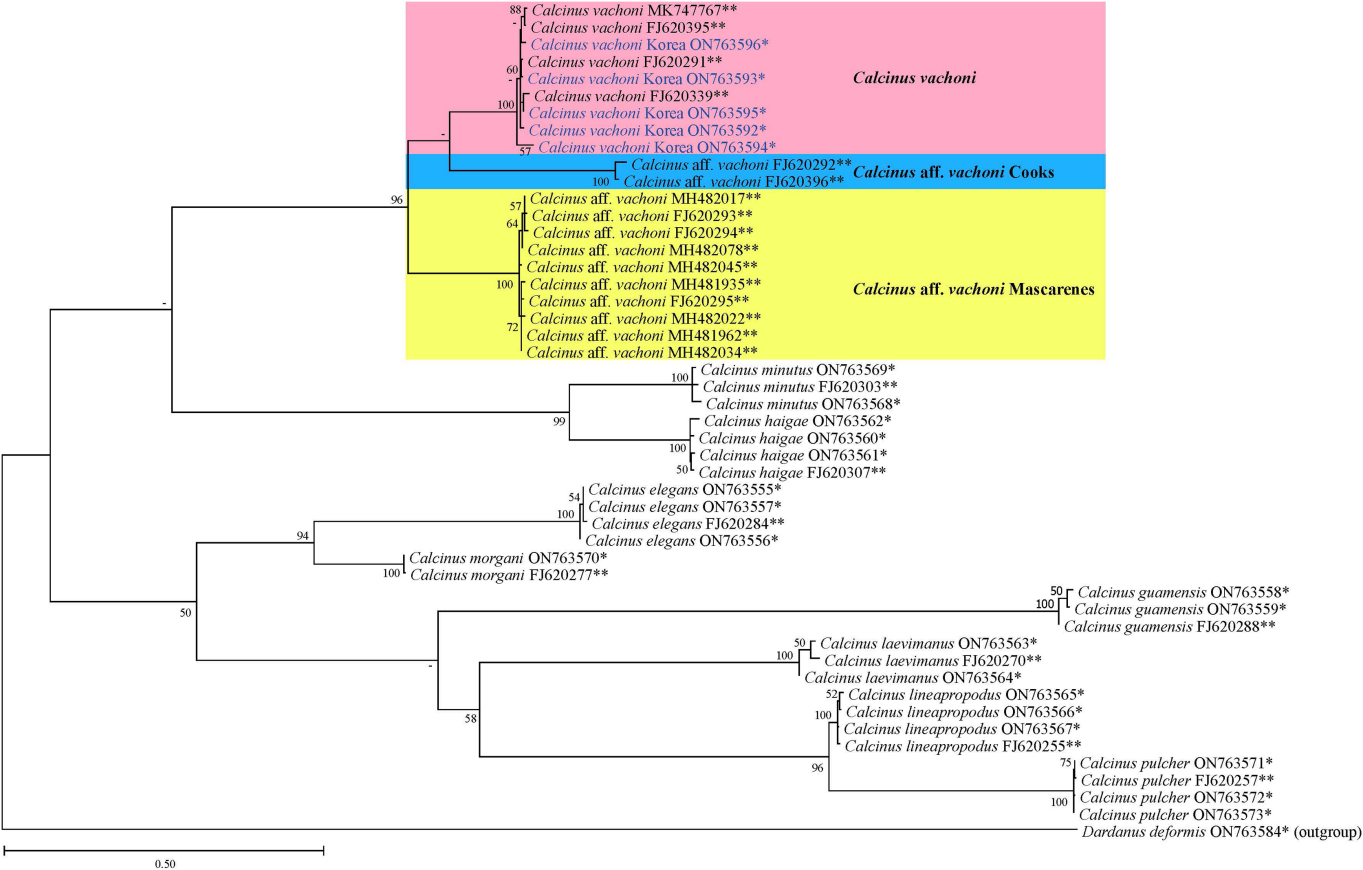
Phylogenetic tree from the maximum likelihood analysis for mtDNA *cox1* sequences of *Calcinus* species, including Korean *C. vachoni* specimens (blue) and *Dardanus deformis* (outgroup). Values on each node indicate bootstrap supporting values (≥50%). The names of the *C. vachoni* haplogroups refer to Malay & Paulay (2010). *Sequences newly determined in this study; **sequences retrieved from GenBank.

**Figure 5:**
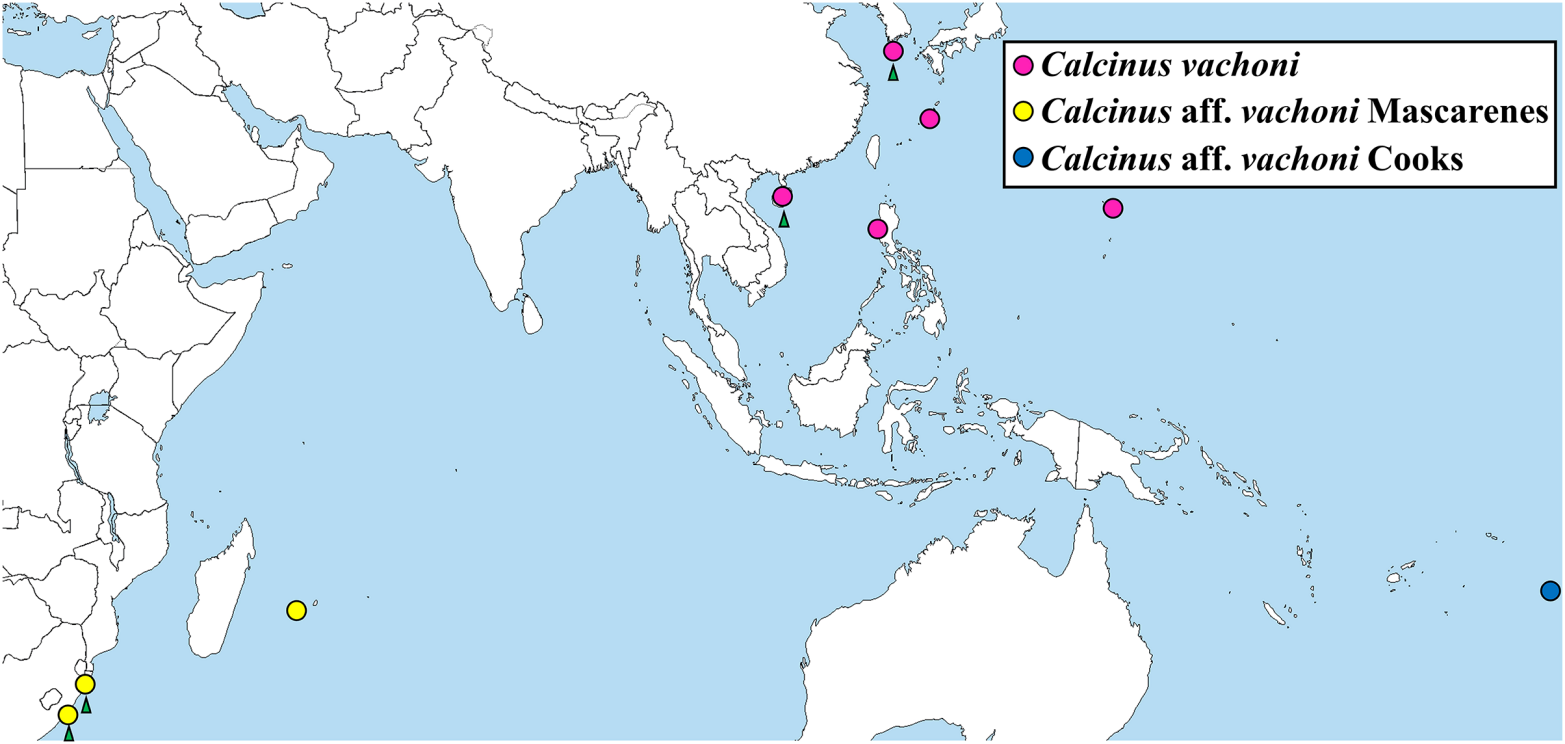
Map showing the localities of *Calcinus* samples whose *cox1* sequences were analyzed in this study. Green arrows: newly identified habitats of the three *C. vachoni* haplogroups in this study.

The phylogenetic tree from ML analysis revealed that *Calcinus* species formed a monophyletic group that is composed of multiple sequence assemblages representing individual *Calcinus* species, each receiving high branch support (mostly ≥99% of bootstrap supporting values; Fig. 4). This result is consistent with the previous phylogenetic study based on multiple molecular markers (*cox1*, 16S and 18S rDNA) that delimited the species boundary of the genus, including *C. vachoni* (Malay & Paulay, 2010). However, interrelationships among *Calcinus* species were not clearly resolved with low supporting values for internal noses in mtDNA *cox1* sequence. Nevertheless, the phylogenetic tree from improved taxon sampling of the Korean *Calcinus* species in this study confirms the previous findings of Malay & Paulay (2010) that the *cox1* sequences of *C. vachoni* were subdivided into three well-supported haplogroups (100% bootstrap support)—*C. vachoni*, *C*. aff. *vachoni* Cooks, and *C*. aff. *vachoni* Mascarenes. This highlights the regional monophyly according to their geographical origin, where the former two haplogroups were more closely related to each other than to *C*. aff. *vachoni* Mascarenes (Figs. 4 and 5). The *C. vachoni* haplogroup sampled from the temperate Northern Pacific/Central Indo-Pacific regions (including the Chinese and Korean *cox1* haplotypes) was initially clustered with the *C*. aff. *vachoni* Cooks haplogroup (with a geographical origin of Cooks Island) belonging to the Eastern Indo-Pacific realm; it was then grouped with *C*. aff. *vachoni* Mascarenes (including the South African *cox1* haplotypes) belonging to the Western Indo-Pacific region. In contrast to the relatively low intra-haplogroup sequence divergence (≤3.19%), the sequence divergence among the three *C. vachoni* haplogroups ranged from 12.2% (between *C. vachoni* and *C*. aff. *vachoni* Mascarenes) to 15.8% (between *C*. aff. *vachoni* Cooks and *C*. aff. *vachoni* Mascarenes); this was comparable to the average sequence divergence (12.3%) between different *Calcinus* species, such as that between *C. haigae* and *C. minutus*. This deep genetic divergence among the three geographic lineages was first discovered by Malay & Paulay (2010) based on the molecular analysis of three gene fragments (*cox1*, 16S rDNA, and H3). According to a recent biogeographic classification of the coastal/shelf areas worldwide that reflects the patterning of marine biodiversity and endemism (Spalding et al., 2007), the three regional haplogroups coincide with their spatial distribution belonging to different marine realms. From morphological and molecular analyses, the results of this study update an extended geographic distribution of *C*. *vachoni* further northward to the southern coast of Korea than previously reported. However, it still remains uncertain whether these three regional haplogroups represent morphologically indistinguishable but genetically distinct cryptic species. To resolve this taxonomic issue (*i.e*., cryptic species complex among different geographical assemblages) and elucidate the underlying mechanisms of their phylogenetic divergence, further investigation is required, including extensive taxon sampling from the three marine biogeographical realms for both morphological and molecular analyses.

### Ecological association between *C. vachoni* and colonial anemones

In this study, we conducted an on-site observation of *C. vachoni* habitat from 12 studied sites in tidepool areas. Out of 12 survey sites, 12 individuals of *C. vachoni* were exclusively found at site #1 where the colonial anemone *P*. aff. *mutuki* co-occurred (Table 2). Although it was estimated from a single event (found at site #1), their co-occurrence with colonial anemones suggests a putative association between *C. vachoni* and *P*. aff. *mutuki*. Hermit crabs are known to have symbiotic associations with many cnidarians, such as hydrozoans, sea anemones, and corals (Williams & McDermott, 2004; Jung & Kim, 2017). In a previous study, *C. vachoni* were reported to inhabit near corals (McLaughlin et al., 2007), and the microhabitat distribution of *C. hazletti* was positively affected by *Pocillopora meandrina*, which is their co-occurring coral species (Hazlett & Bach, 2010). Moreover, Iglesias (2007) suggested that the behavior of *C. latens* (other congeneric species) such as climbing, being withdrawn, being stationary, and locomoting is affected by chemicals released from surrounding organisms. Despite the rich source of information regarding the relationships between hermit crabs and corals, the ecological association between *C. vachoni* and their coral species has not yet been well-characterized. We additionally examined the collection data of *Calcinus* species from Florida Museum Invertebrate Zoology Collection database (http://specifyportal.flmnh.ufl.edu/iz/: as of April 2023) and found that 75% (60 out of 80) of *C. vachoni* records were associated with coral collections (*i.e*., found on dead coral head of *Pocillopora* species), suggesting their occurrence is likely associated with the presence of corals species. High proportion of dead coral heads of *Pocillopora* species provides a rich source of habitat for diverse invertebrates including many crustacean species (Madduppa et al., 2019). Our result is consistent with an earlier report that the occurrence of *C. hazletti* was more predominant in microhabitats of the cauliflower coral *P. meandrina* (Hazlett & Bach, 2010). The presence/absence data of a previous study showed that the behavior of *Calcinus* hermit crabs (*e.g*., *C. latens*) was affected by chemicals released by their co-occurring corals (Iglesias, 2007). Similarly, we observed all *C. vachoni* individuals were found to stay highly stationary with low feeding and locomotory activity until their death within 2 months in the absence of their colonial anemone partner (J Jung, 2021, personal observation). The co-occurrence of two living species does not always represent their ecological interactions (Blanchet, Cazelles & Gravel, 2020). Nevertheless, the implications of the presence/absence data obtained from both on-site and personal observations support the idea that the presence of colonial anemone species *P*. aff. *mutuki* might affect the habitat preference of *C. vachoni* in their natural environments in Korea. The results of this study putatively assume the ecological associations between *C. vachoni* and their colonial anemone counterpart *P*. aff. *mutuki*; however, it has to be validated by compelling evidence from further field observation and experimental studies (*i.e*., *ex situ* study to confirm whether the presence/absence of colonial anemones affects the behavior and survival of the hermit crabs).

## Conclusions

In this study, the tropical hermit crab species *C. vachoni* was first reported in the intertidal zone of Jeju Island—the southernmost island of South Korea. Based on morphological and molecular analyses, we update an extended geographic distribution of *C*. *vachoni* haplogroup further northward to the southern coast of Korea than previously reported. In addition, the present study suggests potential ecological associations between *C. vachoni* and their colonial anemone counterpart *P*. aff. *mutuki*; however, it has to be validated by compelling evidence from further investigation of how the presence/absence of the colonial anemone affects the behavior and survival of the hermit crab *C. vachoni*.

## Additional Information and Declarations

### DNA Deposition

The following information was supplied regarding the deposition of DNA sequences: The cox1 sequences available at GenBank: ON763551 to ON763596, ON763584, and OQ456449--OQ456456.

### Data Availability

The following information was supplied regarding data availability: The data is available at NCBI GenBank: ON763551-ON763596, ON763584, OQ456449--OQ456456. The voucher specimens were deposited in the Honam National Institute of Biological Resources (HNIBR voucher specimen no.: HNIBRIV911). Voucher specimens of Micronesian hermit crabs have been deposited in the Marine Arthropod Depository Bank–MADBK; MADBK voucher specimen nos. are as follows: MADBK 160518_004, MADBK 160518_006, MADBK 160519_016, MADBK 160524_007, MADBK 160530_001, MADBK 160534_004, MADBK 160535_002, MADBK 160536_005, and MADBK 160537_003.
